# ARENA-based activity profiling of tau and TDP-43 transgenic *C. elegans*

**DOI:** 10.17912/micropub.biology.000278

**Published:** 2020-07-16

**Authors:** Heather N Currey, Anna Malinkevich, Penny Melquist, Nicole F Liachko

**Affiliations:** 1 Geriatric Research Education and Clinical Center, Veterans Affairs Puget Sound Health Care System, Seattle, WA 98108 USA; 2 InVivo Biosystems (formerly NemaMetrix, Inc.), Eugene, OR 97402, USA; 3 Division of Gerontology and Geriatric Medicine, Department of Medicine, University of Washington, Seattle, WA 98104, USA

**Figure 1. Activity differences of day 1 adult neurodegenerative disease model C. elegans measured by changes in LED microbeam disruption:**
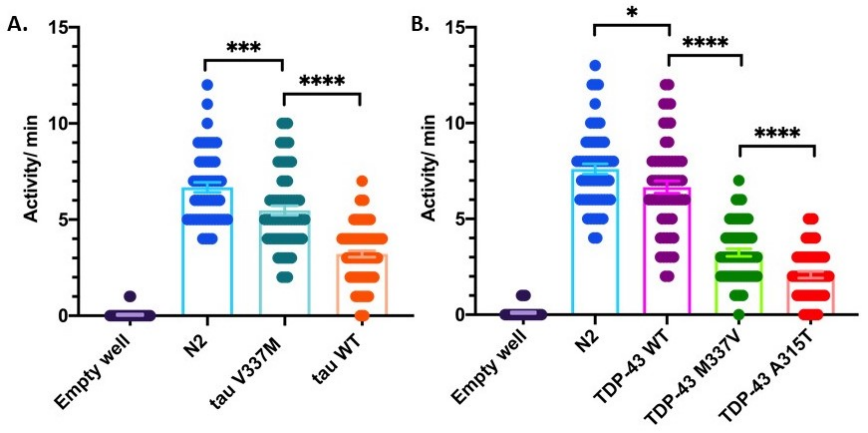
(A) Expression of mutant (tau V337M, strain CK10) or wild-type (tau WT, strain CK144) human tau pan-neuronally causes significant activity differences compared to N2. tau V337M and tau WT are also significantly different from each other. Graph displays aggregate population activity per minute. ***p=0.0005, ****p<0.0001, one-way ANOVA with Tukey’s multiple comparison’s test. Data from 3 independent replicate experiments, N=30 worms per replicate. (B) Expression of wild-type (TDP-43 WT, strain CK410) or mutant (TDP-43 M337V, strain CK423 and TDP-43 A315T, strain CK426) human TDP-43 pan-neuronally causes significant activity differences compared to N2. Each TDP-43 strain was also significantly different from each other. Graph displays aggregate population activity per minute. *p=0.02, ****p<0.0001, one-way ANOVA with Tukey’s multiple comparison’s test. Data from 4 independent replicate experiments, N=30 worms per replicate.

## Description

Aggregates of the protein tau are the hallmark of several neurodegenerative diseases including Alzheimer’s disease, frontotemporal lobar degeneration (FTLD-tau), progressive supranuclear palsy (PSP), corticobasal degeneration (CBD), Pick’s disease, and chronic traumatic encephalopathy (CTE) (VandeVrede, Boxer *et al.* 2020). Mutations in the gene coding for tau, *MAPT*, can cause FTLD-tau, directly linking tau dysfunction with disease (Dickson, Kouri *et al.* 2011). Another protein, TDP-43, comprises aggregates which are the primary hallmark of amyotrophic lateral sclerosis (ALS) and frontotemporal lobar degeneration (FTLD-TDP), and mutations in the gene coding for TDP-43, *TARDBP*, can cause disease (Kawakami, Arai *et al.* 2019). To model tau or TDP-43 proteinopathies, transgenic *C. elegans* have been generated that express the full-length human protein pan-neuronally. These worms exhibit significant uncoordinated movement on plates and impaired thrashing in liquid (Kraemer, Zhang *et al.* 2003; Liachko, Guthrie *et al.* 2010). However, tau- and TDP-43- expressing worms are not paralyzed; they still move their heads and have some motility on the plate (coiling, crawling with tail-drag, head swinging) which are not captured in standard crawling or thrashing assays. To assay differences in total activity, we used a WMicroTracker ARENA System (Phylumtech, AR and InVivo Biosystems, USA). The ARENA captures population level activity data by relying on optical interferometry, which uses a large array of infrared LED microbeams to detect both the movement and position of worms on a culture plate. Disruption of an LED microbeam by worm movement is recorded by repeat scans of the 6-well culture plate, and allows for real-time processing. The software identifies changes in the location of disrupted beams between scans and assigns an activity score based on differences identified between each consecutive scan (Simonetta SH). Both tau- and TDP-43- expressing worms had significantly less activity per minute than N2 (**Figure 1**). Further, we found the ARENA- assessed activity data recapitulated the relative severity of phenotypes among the strains as measured by motility assays. For example, both CK10 (tau V337M) and CK144 (tau WT) have significantly uncoordinated movement when crawling or thrashing in liquid, with CK144 having worse motility than CK10, due to its much higher burden of total tau protein expressed (Kraemer, Zhang *et al.* 2003). Likewise, CK410 (TDP-43 WT) worms have slightly impaired motility compared with N2 when crawling on a plate, CK423 (TDP-43 M337V) are severely uncoordinated, and CK426 (TDP-43 A315T) have the most severe uncoordinated phenotype. The relative toxicities of these strains stem from the effects of the mutations, as TDP-43 protein expression is relatively even among these transgenic strains (Liachko, Guthrie *et al.* 2010). Interestingly, the ARENA captures activity of these severely uncoordinated worms that move poorly in motility assays such as crawling on an NGM plate or thrashing in liquid (Kraemer, Zhang *et al.* 2003; Liachko, Guthrie *et al.* 2010). Therefore, ARENA assessment of aggregate activity may be a more accurate metric for capturing non-locomotor movement of *C. elegans* that are severely uncoordinated.

## Methods

ARENA Tracking

All worms used were staged by way of timed egglay, and grown at 16^o^C on NGM plates seeded with OP50 *E. coli* to day 1 adult. The experiment was performed in a 6-well plate prepared with 3.5mL NGM per well and seeded with OP50. The worms were picked from stock plates into a prepared 6-well plate, 30 worms per strain, each strain in a unique well. Recordings were performed at room temperature (approximately 22^o^C). The plate was placed in the ARENA 15 minutes before starting the run to allow worms to acclimate. 14 activity scores were collected per strain over 60 minutes. Statistical analysis was performed with GraphPad Prism 8.

## Reagents

Strains Used

N2 Bristol

CK10 *bkIs10* [*aex-3p*::*tau(V337M 4R1N);* *myo-2p::dsRED*] III

CK144 *bkIs144* [*aex-3p*::*tau(WT 4R1N);* *myo-2p::dsRED*] IV

CK410 *bkIs410* [*snb-1p*::*TDP-43(WT);* *myo-2p::dsRED*] II

CK423 *bkIs423* [*snb-1p*::*TDP-43(M337V);* *myo-2p::dsRED*] IV

CK426 *bkIs426* [*snb-1p*::*TDP-43(A315T);* *myo-2p::dsRED*] II
